# Muscle Fat and Volume Differences in People With Hip‐Related Pain Compared With Controls: A Machine Learning Approach

**DOI:** 10.1002/jcsm.13608

**Published:** 2024-09-29

**Authors:** Chris Stewart, Evert O. Wesselink, Zuzana Perraton, Kenneth A. Weber, Matthew G. King, Joanne L. Kemp, Benjamin F. Mentiplay, Kay M. Crossley, James M. Elliott, Joshua J. Heerey, Mark J. Scholes, Peter R. Lawrenson, Chris Calabrese, Adam I. Semciw

**Affiliations:** ^1^ School of Allied Health, Human Services and Sport, Discipline of Physiotherapy La Trobe University Melbourne Australia; ^2^ Faculty of Behavioural and Movement Sciences, Amsterdam Movement Sciences Vrije Universiteit Amsterdam Amsterdam The Netherlands; ^3^ Department of Anesthesiology, Perioperative and Pain Medicine Stanford University Palo Alto California USA; ^4^ La Trobe Sport and Exercise Medicine Research Centre, School of Allied Health, Human Services and Sport La Trobe University Bundoora Australia; ^5^ Discipline of Sport and Exercise Science, School of Allied Health, Human Services and Sport La Trobe University Melbourne Australia; ^6^ Faculty of Medicine and Health, Northern Sydney Local Health District and the University of Sydney The Kolling Institute St Leonards Sydney Australia; ^7^ School of Health and Rehabilitation Sciences University of Queensland Brisbane Australia; ^8^ Innovation and Research Centre, Community and Oral Health Directorate Metro North Health Brisbane Australia; ^9^ Department of Allied Health Northern Health Epping Australia

**Keywords:** Buttocks, Gluteal, Muscle, Pain

## Abstract

**Background:**

Hip‐related pain (HRP) affects young to middle‐aged active adults and impacts physical activity, finances and quality of life. HRP includes conditions like femoroacetabular impingement syndrome and labral tears. Lateral hip muscle dysfunction and atrophy in HRP are more pronounced in advanced hip pathology, with limited evidence in younger populations. While MRI use for assessing hip muscle morphology is increasing, with automated deep‐learning techniques showing promise, studies assessing their accuracy are limited. Therefore, we aimed to compare hip intramuscular fat infiltrate (MFI) and muscle volume, in individuals with and without HRP as well as assess the reliability and accuracy of automated machine‐learning segmentations compared with human‐generated segmentation.

**Methods:**

This cross‐sectional study included sub‐elite/amateur football players (Australian football and soccer) with a greater than 6‐month history of HRP [*n* = 180, average age 28.32, (standard deviation 5.88) years, 19% female] and a control group of sub‐elite/amateur football players without pain [*n* = 48, 28.89 (6.22) years, 29% female]. Muscle volume and MFI of gluteus maximus, medius, minimis and tensor fascia latae were assessed using MRI. Associations between muscle volume and group were explored using linear regression models, controlling for body mass index, age, sport and sex. A convolutional neural network (CNN) machine‐learning approach was compared with human‐performed muscle segmentations in a subset of participants (*n* = 52) using intraclass correlation coefficients and Sorensen–Dice index.

**Results:**

When considering adjusted estimates of muscle volume, there were significant differences observed between groups for gluteus medius (adjusted mean difference 23 858 mm^3^ [95% confidence interval 7563, 40 137]; *p* = 0.004) and tensor fascia latae (6660 mm^3^ [2440, 13 075]; *p* = 0.042). No differences were observed between groups for gluteus maximus (18 265 mm^3^ [−21 209, 50 782]; *p* = 0.419) or minimus (3893 mm^3^ [−2209, 9996]; *p* = 0.21). The CNN was trained for 30 000 iterations and assessed its accuracy and reliability on an independent testing dataset, achieving high segmentation accuracy (mean Sorenson–Dice index >0.900) and excellent muscle volume and MFI reliability (ICC_2,1_ > 0.900). The CNN outperformed manual raters, who had slightly lower interrater accuracy (Sorensen–Dice index >0.800) and reliability (ICC_2,1_ > 0.800).

**Conclusions:**

The increased muscle volumes in the symptomatic group compared with controls could be associated with increased myofibrillar size, sarcoplasmic hypertrophy or both. These changes may facilitate greater muscular efficiency for a given load, enabling the athlete to maintain their normal level of function. In addition, the CNNs for muscle segmentation was more efficient and demonstrated excellent reliability in comparison to manual segmentations.

## 1 Introduction

Hip‐related pain (HRP) commonly affects young to middle‐aged active adults (18–50 years) and has significant implications for physical activity, financial responsibilities and overall quality of life [1]. HRP encompasses conditions such as femoroacetabular impingement syndrome (FAIs), hip dysplasia and intra‐articular pathologies like labral tears, chondral damage and ligamentum teres tears without distinct osseous morphology [2].

The lateral hip muscles, including the gluteus maximus (Gmax), gluteus medius (Gmed), gluteus minimus (Gmin) and tensor fasciae latae (TFL), play crucial roles in hip joint stability and movement during various functional tasks such as walking and stepping [3]. Gmed, Gmin and TFL contribute to frontal plane stability of the pelvis and femur during walking [4], while Gmax provides stability in the sagittal and transverse planes, acting as a hip extensor and rotator [3]. Lateral hip muscle dysfunction and atrophy appear to be muscle‐specific and more affected in those with advanced hip joint pathology or symptoms [5]; however, the evidence is limited in those from a younger population with HRP.

The use of magnetic resonance imaging (MRI) to assess the lateral hip muscle volume and quality is exponentially increasing [6]. A limitation previously has been the time and resource‐intensive nature of manually tracing muscles from the MRI images. Recent advances in MRI technology include the development of automated deep‐learning tracing techniques through machine learning [7]. Automated techniques and analysis through machine learning are more time efficient than manual tracing, reducing analysis time from hours to seconds while maintaining near human‐level performance [8]. Manual methods for tracing muscles for size and intramuscular fat infiltrate (MFI) are currently the gold standard, with limited studies assessing the accuracy of automated methods in lateral hip muscles [7]. Therefore, the primary aim of this study was to compare MFI and muscle volume in individuals with HRP and asymptomatic controls. The secondary aim was to assess the reliability and accuracy of automated machine‐learning segmentations compared with human‐generated segmentation.

## 2 Methods

### 2.1 Study Design

This cross‐sectional study used baseline data from the femoroacetablar impingement and hip OsteoaRthritis Cohort (FORCe) study [9]. The study was approved by the La Trobe University Human Ethics Committee (HEC15‐019 and HEC16‐045) and the University of Queensland Medical Research Ethics Committee (No. 2015000916:2016001694); participants provided written informed consent before participating.

### 2.2 Participants

The study recruited sub‐elite/amateur football (Australian Football and soccer) players with a greater than 6‐month history of HRP (*n* = 182) and a control group of sub‐elite/amateur footballers without such pain (*n* = 50) [9]. Recruitment was carried out through various methods, including social media advertising, sporting event stalls, posters and direct contact with clubs around the greater metropolitan Melbourne and Brisbane region. The recruitment period spanned from August 2015 to October 2018.

A detailed overview of the study's inclusion and exclusion criteria is outlined in Table S1. In brief, football players were eligible to participate if they were aged between 18 and 50 years old, participated in a minimum of two sessions of football (games or training) per week and did not have signs of radiographic osteoarthritis (defined as a Kellgren and Lawrence score of ≥2) on an anterior–posterior pelvis radiograph. For the symptomatic group, football players had to present with a minimum 6‐month history of insidious onset of hip and/or groin pain, report impingement‐type symptoms (e.g., pain with kicking, squatting and change of direction) and have a positive flexion adduction and internal rotation (FADIR) test. For the control group, they had to present with no self‐reported history of hip and or groin pain, a negative FADIR test, and no history of lower‐limb surgery.

### 2.3 Participants in the Machine Learning Subset

A subset of participants was used to train and test a convolutional neural network (CNN) model for the automated segmentation of the lateral hip muscles. A consecutive sample of pelvic and hip MRI datasets from 52 participants were included in this subset, with 28 participants from the asymptomatic group and 24 from the symptomatic group.

## 3 Data Collection

### 3.1 Patient‐Reported Outcome Measures

During data collection, detailed demographic data were gathered, including variables such as sex, age, height, body mass, football code and dominant kicking foot (lower‐limb dominance). Participant HRP burden was quantified via the International Hip Outcome Tool 33 question addition (iHOT‐33). The iHOT‐33 has been deemed valid and reliable in football players with HRP and is recommended for use in young to middle‐aged individuals, consistent with those recruited in the study [10, 11].

### 3.2 MRI Evaluation

A 3.0‐Tesla MRI (Philips Ingenia, Netherlands) was used to assess hip muscle volume and MFI following a standard protocol [12]. Participants were positioned supine and feet first with a 32‐channel torso coil placed over the hips and pelvis. High‐resolution fat and water images of the pelvis were acquired with the field‐of‐view (FOV) (270 mm × 380 mm) spanning from the iliac crest to the upper femur (slice thickness, 1.5 mm; slice gap, 0 mm; repetition time, 3.5 ms; echo time (TE_1_), 1.2 ms; echo time (TE_2_), 2.3 ms, in plane resolution of 1.5 mm × 1.5 mm; bandwidth 1286 Hz, number of slices = 216). Multi‐echo Dixon images were acquired, which gives superior separation of fat for quantification [13]. MRI images were carefully reviewed, and images with observable artefacts were excluded from the analysis.

### 3.3 Convolutional Neural Network (CNN) Training and Testing

**FIGURE 1:**
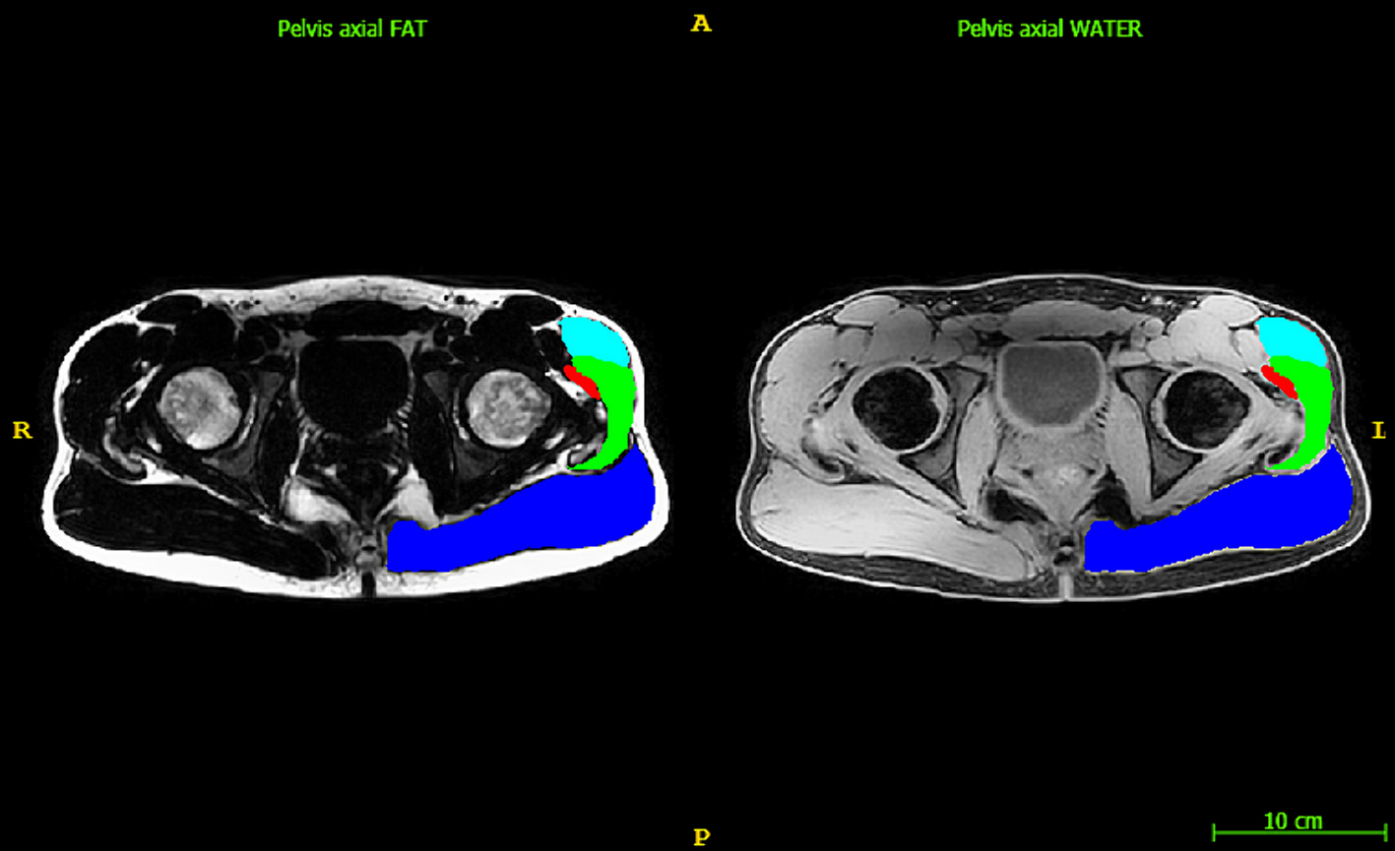
Multi‐echo DIXON fat and water images. Pelvic axial slice at the level of the most superior tip of the greater trochanter. Left fat image, right water image. Segmentations were performed on both the left and right sides; however, only the left side is presented here. Left lateral hip muscles segmented: Blue, gluteus maximus; green, gluteus medius; red, gluteus minimus; aqua; tensor fascia latae. A, anterior; L, left; P, posterior; R, right.

A blinded rater manually segmented the Gmax, Gmed, Gmin and TFL from the water images of 24 and 28 symptomatic and asymptomatic participants, respectively, using ITK‐snap (version 3.6) [14] (Figure 1). The left and right muscles were segmented separately. We then split the images into training (*n* = 30), validation (*n* = 8) and testing (*n* = 14) datasets. The CNN sample sizes are similar to previous reports and were chosen to reduce the burden of the time‐consuming manual segmentation while providing sufficient clinical variability in the data to ensure generalizability of the model [8, 15]. We trained a 3D U‐Net CNN architecture (activation unit = PReLU; channels = 16, 32, 63, 128 and 256; spatial window size = 336 × 336 × 32; strides = 2, 2, 2 and 2; number of residual units = 2; and normalization = batch) to perform multiclass segmentation using the water images as features and the manual segmentations as the ground truth. The CNN was trained on a NVIDIA RTX 3070 24GB graphical processing unit (CUDA version = 12.1, Santa Clara, CA) (optimizer = AdamW; loss function = DiceCEloss, weight decay = 1 × 10^−5^, learning rate = 1 × 10^−4^ and batch size = 1). We assessed the validation accuracy every 500 iterations using the Sorensen–Dice index (Dice) and trained the model until the segmentation performance plateaued on the validation dataset. Model training and inference were performed using the open‐source MONAI Python package (version = 0.9) based on PyTorch (version = 1.10.2). We have used a similar approach to previously published methods of automatic segmentation of musculature [15].

Inference was performed on the testing dataset using the trained CNN. We then assessed segmentation accuracy for each muscle with respect to the ground truth using the Dice index, Jaccard index (JI), conformity coefficient (CC), true positive rate (TPR), true negative rate (TNR), positive predictive value (PPV) and volume ratio (VR) measures. For the purpose of assessing the reliability and accuracy of the CNN model, the mean MFI across the entire muscle was calculated for each muscle. We further assessed the accuracy and reliability of the CNN with respect to the ground truth using Bland–Altmann plots, Pearson correlations, mean absolute error (MAE), root mean squared error (RMSE), coefficient of determination (*R* ^2^) and intraclass correlation coefficients (ICC_2,1_) for muscle volume and MFI.

### 3.4 Interrater Reliability

To compare the CNN accuracy and reliability to manual segmentation, another blinded independent rater manually segmented a subset of the images (*n* = 36), and we used the same approach and measures to assess interrater reliability. Both raters (C. S. and C. C.) were registered Australian physiotherapists with extensive hip anatomy and imaging training. Analysis was performed on the entire data set of the 182 files, and 50 controls were performed with visual inspection and manual correction as necessary.

#### 3.4.1 Image Acquisition and Analysis

##### 3.4.1.1 Volume

To calculate the volume (mm^3^), the cross‐sectional area (CSA) (mm^2^) of each axial slice was obtained using the ‘neurobase’ (version 1.32.3) R package (version 2022.2.2.485) and multiplied by slice thickness (mm).

##### 3.4.1.2 Fat

The MFI was calculated to represent the proportion of fat to total muscle volume [fat/(fat + muscle)]. Customized scripts in R were used to calculate muscle‐fat index for each slice along the length of the muscle. These values were then normalized to 101 data points along the length of the muscle using spline interpolation so that the muscle‐fat index could be represented at each percentage of the muscle length from proximal (0%) to distal (100%) as done previously [16]. Given the segmental nature of Gmin [17] and Gmed [18], this value was determined for three separate segments of Gmed (anterior, middle and posterior) and two separate segments of Gmin (anterior and posterior).

### 3.5 Statistical Analysis

For the analysis, one limb from each participant was selected. Participants reported their most symptomatic limb based on their responses to the question, ‘which hip gives you the most trouble?’ [19]. To determine which hip was used for analysis in the asymptomatic participants, the distribution of kicking versus non‐kicking limbs in the symptomatic group was calculated, stratified by sex. The testing limb of the asymptomatic group was then established by using a random line shuffle to match the gender‐stratified distribution [20, 21].

MFI along the length of the muscle was described narratively to define peak areas of fat. Muscle fat was then compared between the symptomatic and asymptomatic participants using a two‐sample *t*‐test within the statistical parametric mapping (SPM) package (spm1D v0.4, http://www.spm1D.org), conducted in Python 2.7. The alpha level was set at 0.05, and the SPMs and the critical‐*t* value were calculated along the length of the muscle. The muscle fat variable of interest was analysed, and the two groups were considered significantly different when the SPM exceeded the calculated critical *t*‐value. The difference in muscle fat helped determine the disparity between the symptomatic and control groups over any point along the length of the muscle.

A linear regression model was employed to examine the between‐group difference in muscle volume. Initially, we assessed for an interaction between symptomatic status and sex. If the *p*‐value for the interaction term was greater than 0.05, indicating a lack of significant interaction, the interaction term was removed from the model. All analyses were adjusted for age, BMI, sport (Australian Football or soccer) and sex to account for their potential confounding effects on the relationship between muscle volume and symptomatic status.

### 3.6 Results

From the 182 available participants in the symptomatic group, two participants were excluded due to corrupt files that could not be corrected. Out of the 52 asymptomatic participants available for the study, two individuals were excluded due to MRI artefacts, and an additional two participants had incomplete data. The age, height, body mass and BMI for each group are presented in Table 1.

**TABLE 1:** Participant demographics.

	Hip‐related pain group	Control group
Number of participants	*N* = 180	*N* = 48
	(*F* = 37, 19%)	(*F* = 14, 29%)
Age (year)	28.32 (5.88)	28.89 (6.22)
Height (cm)	178 (9)	177 (10)
Body mass (kg)	78.63 (12.64)	76.78 (13.75)
BMI (kg/m^2^)	24.67 (3.17)	24.27 (3.12)
iHOT33 total	66.23 (17.69)	97.21 (3.86)
Sport	AF = 88 (49%)	AF = 20 (42%)
	Soccer 92 (51%)	Soccer 28 (58%)

*Note:* Data reported as mean.

Abbreviations: AF, Australian football; BMI, body mass index; F, female; iHOT‐33, International Hip Outcome Tool – 33 question edition; SD, standard deviation.

### 3.7 CNN Accuracy and Reliability

We trained the CNN for 30 000 iterations. We then assessed the accuracy and reliability of the trained CNN on the independent testing dataset (*n* = 14) for each muscle separately. Overall, CNN segmentation accuracy was high (Table S2). The mean Dice index was >0.900 for all muscles, highest for GMax (≈0.960) and lowest for GMin (≈0.900). Muscle volume also demonstrated high accuracy and reliability. The volume error was <10% of the total muscle volume across all error measures for each muscle. MFI accuracy was also high with MFI error <2% across all error measures for every muscle except the TFL where the RMSE was 2.6% and 2.4% for the left and right TFL, respectively. The reliability of muscle volume and MFI was excellent (ICC_2,1_ > 0.900) for all muscles. The interrater accuracy and reliability between two manual raters were lower than the CNN. The two manual raters had high interrater accuracy (Sorensen–Dice index > 0.800) and good to excellent reliability (ICC_2,1_ > 0.800) for all muscles (Tables S2 and S3, Figures S1 and S2).

#### 3.7.1 Intramuscular fat Infiltrate

**FIGURE 2:**
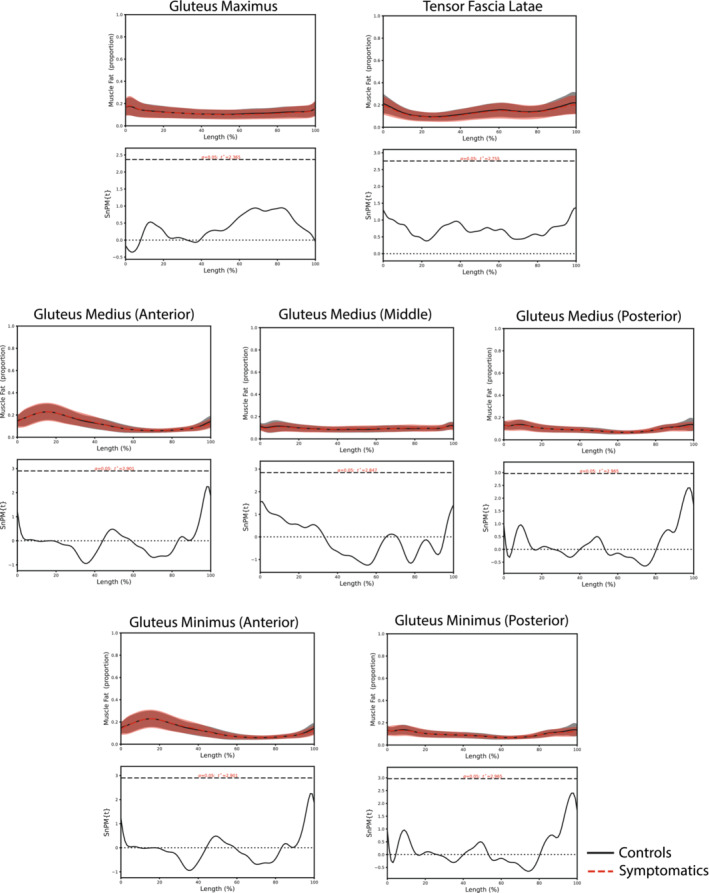
Muscle fat differences between symptomatic (red) and control (black).

The distribution of MFI was found to be non‐uniform along the muscle length for both the symptomatic and asymptomatic groups (Figure 2). The Gmax muscle showed increased MFI proximally and distributed evenly throughout the muscle's length. The anterior GMed and GMin muscles exhibited a high proportion of MFI in the proximal third of the muscle. The posterior Gmin and Gmed as well as middle Gmed showed an even distribution of MFI throughout the muscle's length, while the TFL muscle showed an increase in MFI in the distal end. There was no significant difference in muscle fat infiltration between the symptomatic and control groups for all the lateral hip muscles (Figure 2).

#### 3.7.2 Muscle Volume

**FIGURE 3:**
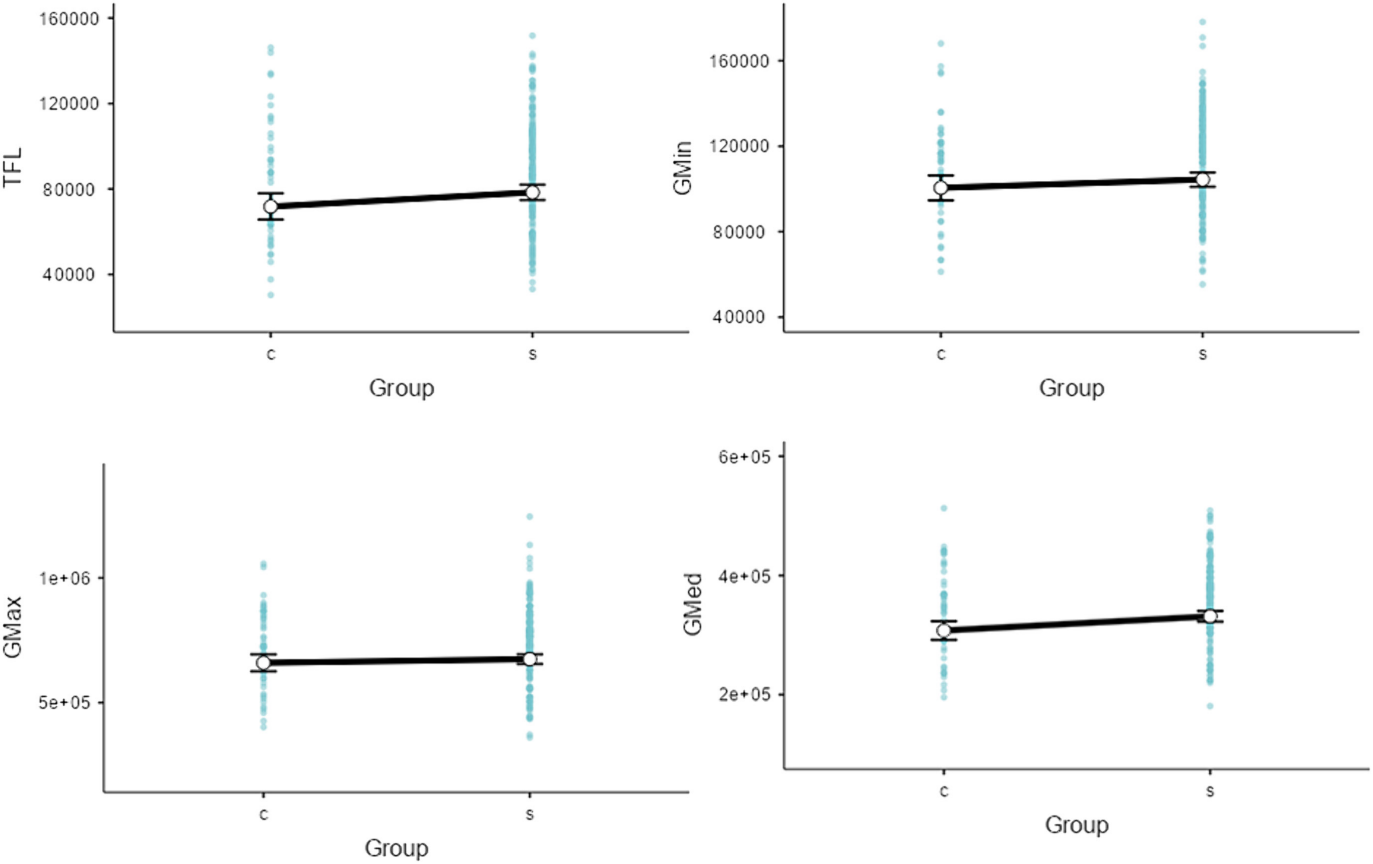
Mean (95% CI) muscle volume of control (c) and symptomatic (s) participants.

There was no interaction between symptomatic status and sex on any of the muscles examined [Gmax (*p* = 0.49), Gmed (*p* = 0.47), Gmin (*p* = 0.47) and TFL (*p* = 0.34)] (Figure 3).

When considering adjusted estimates of muscle volume, there were significant differences observed between the symptomatic and asymptomatic groups for Gmed (adjusted mean difference 23 858 mm^3^ [95% confidence interval 7563, 40 137]; *p* = 0.004) and TFL (6660 mm^3^ [2440, 13 075]; *p* = 0.042). This indicates a larger volume in the symptomatic group for the Gmed (7.8% larger) and TFL (9.3%) muscles compared with the asymptomatic group. No differences were observed between the symptomatic and asymptomatic groups for Gmax (18 265 mm^3^ [−21 209, 50 782]; *p* = 0.419) and Gmin (3893 mm^3^ [−2209, 9996]; *p* = 0.21).

## 4 Discussion

This study compared the MFI and volume of the lateral hip muscles in football players with and without HRP. The results revealed no significant differences in MFI between the two groups. Adjusted estimates revealed greater muscle volume in symptomatic compared with asymptomatic groups for Gmed and TFL. No significant differences were found for Gmax and Gmin volume. MFI distribution of the lateral hip muscles did not differ between the groups and was consistently variable along the muscle length. The use of CNN for muscle segmentation exhibited high accuracy, with mean Sorensen–Dice index exceeding 0.900 for all muscles. Muscle volume and MFI segmentation by CNN were reliable, with minimal errors.

### 4.1 Muscle Volume

The present study observed an increase in muscle volume, specifically in the Gmed and TFL, in the symptomatic group compared with the asymptomatic group. These findings are consistent with previous studies reporting increased hip muscle volume in symptomatic individuals experiencing HRP [22] and early‐stage or mild hip OA, where an increase in volume was found in Gmed compared with asymptomatic individuals [23]. One limitation of previous research is that the gluteal muscles (Gmin and Gmed) have been combined [22]. It is important to note that, in our study, the increase in muscle volume is unlikely to be attributed to an increase in MFI, as this study did not find any evidence of a greater proportion of muscle fat. One plausible contributing factor could be the presence of pain, which may induce an adaptive response resulting in increased muscle volume [23]. There is evidence of a loss in frontal plane range of motion when walking in people with HRP [24], which may be influenced primarily by lower hip abduction angle during stance [25]. In a study by Grimaldi et al. [23], it was proposed that an increase in muscle volume could be associated with compensatory mechanisms to overcome deficiencies in hip strength. The hypothesis was that the increased volume of the Gmed muscle may result from the muscle's effort to provide a more effective length‐tension response. Such a compensatory mechanism could feasibly improve the function and stability of the hip joint in this population.

### 4.2 Intramuscular Fat Infiltrate

In the early stages of hip pain, our study indicates no difference in gluteal muscle fat between individuals with HRP and controls [26]. Compared with their matched peers, the elevated levels of gluteal muscle fat observed in individuals with advanced hip OA suggest that the accumulation of muscle fat may serve as a compensatory response to pain and/or symptoms [27]. The exact time‐course of these changes requires further work with prospective cohort studies.

Regardless of symptoms, we did observe notable variations in MFI distribution along the anterior and middle regions of Gmed and the anterior region of the Gmin muscle. Higher proportions of MFI were observed within the proximal and distal thirds of these muscles. The significance of these high‐fat content localized regions is poorly understood. It is believed that the variability in muscle fat content may partly explain altered muscle composition and biomechanics, influencing clinical outcomes and potentially affecting the transition from acute to chronic musculoskeletal disorders [13]. With this in mind, it is pertinent to acknowledge that our participants might have been in the early stages of HRP, where associated impairments might not have been substantial enough to detect observable changes in MFI. This underscores the necessity for further exploration into the progression of MFI at distinct stages along the trajectory of hip pain.

### 4.3 Automating Muscle Segmentation

The utilization of CNN's for muscle segmentation is notably more efficient and time‐effective compared with the manual tracing of muscles. What typically takes 4 to 8 h through manual tracing can now be accomplished within seconds. This approach has been successfully implemented in other anatomical regions, demonstrating high accuracy and reliability in providing objective muscle volume measurements and MFI [8, 28]. It is important to note that a crucial step involves performing quality checks on the segmented data to identify and rectify any potential segmentation errors. This quality control step is typically much faster and more efficient than the time required for manual segmentation, usually taking only 10 to 20 min, depending on the extent of manual adjustments needed.

The CNNs high accuracy allows for the processing and analysis of larger data sets, resulting in greater power to detect differences or associations and more objective results from the automated method. The CNN saves a substantial amount of time, which then leads to more time for analysing and interpreting results. Using CNN's paves the way for clinical translation of these techniques, which is impossible with manual segmentation. Before the clinical translation of these methods, the clinical utility needs to be further demonstrated, including establishing clinical cut‐offs that aid in the clinical decision‐making of HRP. We hope to further inform clinical translation by updating our CNN toolbox in due course https://github.com/MuscleMap.

### 4.4 Limitations

There are limitations associated with this study that require acknowledgement. Firstly, this study focused on sub‐elite football players, which may not provide a true representation of the general population in terms of the relationship between MFI and lateral hip muscle volume. The study also exclusively included participants with long‐standing HRP lasting 6 months or more, making it challenging to generalize the results to acute cases. Future studies should aim to address these limitations and include a more diverse sample from the general population to obtain a broader understanding of the association between muscle fat infiltration and hip volume. The CNN model was only trained on a single contrast and set of imaging parameters. It is unknown how well the model will generalize to other MRI datasets.

### 4.5 Clinical Implication

The increased muscle volumes in the symptomatic groups compared with controls, particularly in Gmed and TFL, is likely a compensatory mechanism in response to pain. These changes could be associated with increased myofibrillar size, sarcoplasmic hypertrophy or both [29]. These compensatory changes may facilitate greater muscular efficiency for a given load, enabling the patient to maintain their normal level of function. Clinicians may be advised to augment these strategies, by offering solutions to further reduce symptoms, while maintaining function. Potential suggestions could include the use of foot orthoses [30, 31], or gait modification techniques which may include addressing stride length [32] or promoting greater foot/ankle function while walking (e.g., ‘push more with your feet when you walk’) [33]. We also know that the increased muscle size observed in this study acutely may be short lived. We should encourage exercise interventions to reduce the impact of pain on future muscle wasting.

The need for prospective studies that track changes over time is crucial in understanding the progression of muscle morphology and MFI in HRP. By following participants longitudinally, researchers can identify specific time points where muscle changes may occur. Further data from the FORCE cohort [9] will hopefully address these important concepts.

## 5 Conclusion

Our study contributes significant insights into the relationship between hip muscle volume and MFI in individuals with HRP compared with asymptomatic individuals. Specifically, we observed increased muscle volume in the Gmed and TFL muscles during the early stages of HRP. This mechanism may enhance the hip joint's function and stability in response to the presence of HRP.

## Disclosure

The content is solely the responsibility of the authors and does not necessarily represent the official views of the National Institutes of Health. Funding sources had no role in study design, collection, analysis, and interpretation of data, writing the report, or in the decision to submit the article for publication.

## Ethics Statement

La Trobe University Human Ethics Committee (HEC 15‐019 and HEC 16‐045) and the University of Queensland Human Ethics Committee (2015000916 and 2016001694).

## Conflicts of Interest

The authors declare no conflicts of interest.

## Supporting information


**Table S1.** Inclusion and exclusion criteria for participants.
**Table S2.** CNN Segmentation Metrics.
**Table S3.** Testing Volume and MFI Accuracy and Reliability.
**Figure S1.** Reliability and accuracy of the convolutional neural network (CNN) volume measures (ml) with respect to the ground truth (GT) on the testing dataset (*n* = 14). Correlation and Bland–Altman plots are shown for each of the muscles. In the correlation plots, the solid black line represents the best fit line, and the dashed grey line represents perfect prediction (CNN = GT). In the Bland–Altman plots, the dashed black and grey lines indicate the bias (mean error) and the 95% limits of agreement (mean error ± 1.96 × standard deviation), respectively.
**Figure S2.** Reliability and accuracy of the convolutional neural network (CNN) muscle fat infiltration (MFI) measures (%) with respect to the ground truth (GT) on the testing dataset (*n* = 14). Correlation and Bland–Altman plots are shown for each of the muscles. In the correlation plots, the solid black line represents the best fit line, and the dashed grey line represents perfect prediction (CNN = GT). In the Bland–Altman plots, the dashed black and grey lines indicate the bias (mean error) and the 95% limits of agreement (mean error ± 1.96 × standard deviation), respectively.
