# Supplementary material for: Muscle Fat and Volume Differences in People With Hip‐Related Pain Compared With Controls: A Machine Learning Approach
Source: J Cachexia Sarcopenia Muscle. 2024 Sep 29;15(6):2642–50. doi: 10.1002/jcsm.13608 (PMC11634488; doi:10.1002/jcsm.13608)
Supplement: Supplementary file 1 — Table S1. Inclusion and exclusion criteria for participants. Table S2. CNN Segmentation Metrics. Table S3. Testing Volume and MFI Accuracy and Reliability. Figure S1. Reliability and accuracy of the convolutional neural network (CNN) volume measures (ml) with respect to the ground truth (GT) on the testing dataset (n = 14). Correlation and Bland–Altman plots are shown for each of the muscles. In the correlation plots, the solid black line represents the best fit line, and the dashed grey line represents perfect prediction (CNN = GT). In the Bland–Altman plots, the dashed black and grey lines indicate the bias (mean error) and the 95% limits of agreement (mean error ± 1.96 × standard deviation), respectively. Figure S2. Reliability and accuracy of the convolutional neural network (CNN) muscle fat infiltration (MFI) measures (%) with respect to the ground truth (GT) on the testing dataset (n = 14). Correlation and Bland–Altman plots are shown for each of the muscles. In the correlation plots, the solid black line represents the best fit line, and the dashed grey line represents perfect prediction (CNN = GT). In the Bland–Altman plots, the dashed black and grey lines indicate the bias (mean error) and the 95% limits of agreement (mean error ± 1.96 × standard deviation), respectively. [file JCSM-15-2642-s001.docx]

**SUPPLEMENTARY MATERIAL**

**Supplementary Table:** 1 Inclusion and exclusion criteria for participants

| Inclusion criteria | Exclusion criteria |
| --- | --- |
| **Symptomatic**  -Men and women aged 18 – 50 years  -Playing in at least two football and/or soccer sessions per week (training or matches).  -Minimum 6-month history of insidious-onset of hip and/or groin pain.  -Positive FADIR test  -Subjective report of impingement-type symptoms, for example:   - Clicking. - Locking. - Catching. - Giving away. - Pain with sitting, squatting, kicking, cutting/change of direction. | -History of any significant hip or groin condition/ trauma, self-reported as diagnosed by a registered health professional (e.g. doctor or physiotherapist), Specifically:   - Acute hip or groin injury. - Rheumatoid arthritis. - Congenital dislocation of the hip/development dysplasia. - Perthes disease. - Slipped upper femoral epiphysis. - Subluxation and dislocations. - Fracture. - Septic arthritis. - Bursitis (gluteal or adductor). - Acute groin strain.   -Non-insidious onset of pain.  -Positive FADIR test on contralateral hip.  -Previous hip and/or pelvis surgery.  -Pain in the lumbar spine or lower limb in the previous three months  -Intra-articular injection into the hip in the previous three months.  -Contraindication to radiography/MRI  -Kellegren & Lawrence grade ≥2 on AP pelvis Xray  -Unable to understand spoken or written English. |
| **Asymptomatic**  -Men and Women aged 18 - 50 years  -Playing in at least two sessions of football and/or soccer per week (training or matches).  -Negative testing on FADIR | -No History of self-reporting:   - Hip and groin pain - Hip or groin condition (same exclusion criteria for symptomatic group) - Previous lower limb surgery. - Lumbar spine or lower limb in the last three months, and unable to bear weight through the lower limbs.   -Unable to understand English  -Kellegren & Lawrence ≥2 on AP pelvis xray |
| FADIR=Flexion, adduction, internal rotation. | |

**Supplementary Table 2** CNN Segmentation Metrics

CNN Segmentation Metrics

| Muscle | Dice | JI | CC | TPR | TNR | PPV | VR |
| --- | --- | --- | --- | --- | --- | --- | --- |
| GMin | | | | | | | |
| Left | 0.903 ± 0.004 | 0.824 ± 0.007 | 0.786 ± 0.010 | 0.935 ± 0.007 | 1.000 ± 0.000 | 0.875 ± 0.008 | 1.071 ± 0.016 |
| Right | 0.906 ± 0.004 | 0.829 ± 0.006 | 0.793 ± 0.009 | 0.915 ± 0.008 | 1.000 ± 0.000 | 0.899 ± 0.006 | 1.019 ± 0.014 |
| GMed | | | | | | | |
| Left | 0.941 ± 0.003 | 0.889 ± 0.005 | 0.875 ± 0.006 | 0.947 ± 0.006 | 1.000 ± 0.000 | 0.936 ± 0.006 | 1.013 ± 0.012 |
| Right | 0.935 ± 0.006 | 0.878 ± 0.010 | 0.859 ± 0.015 | 0.939 ± 0.013 | 1.000 ± 0.000 | 0.933 ± 0.008 | 1.008 ± 0.019 |
| GMax | | | | | | | |
| Left | 0.966 ± 0.001 | 0.935 ± 0.002 | 0.930 ± 0.003 | 0.968 ± 0.003 | 0.999 ± 0.000 | 0.965 ± 0.002 | 1.003 ± 0.005 |
| Right | 0.964 ± 0.002 | 0.931 ± 0.003 | 0.926 ± 0.004 | 0.959 ± 0.004 | 0.999 ± 0.000 | 0.970 ± 0.002 | 0.989 ± 0.006 |
| TFL | | | | | | | |
| Left | 0.939 ± 0.004 | 0.886 ± 0.008 | 0.870 ± 0.010 | 0.922 ± 0.009 | 1.000 ± 0.000 | 0.958 ± 0.006 | 0.964 ± 0.013 |
| Right | 0.941 ± 0.004 | 0.890 ± 0.008 | 0.875 ± 0.010 | 0.930 ± 0.008 | 1.000 ± 0.000 | 0.954 ± 0.004 | 0.976 ± 0.010 |

Supplementary Table 2. CNN segmentation metrics. CNN accuracy with respect to the ground truth was assessed using the Sørensen-Dice index (Dice), Jaccard index (JI), conformity coefficient (CC), true positive rate (TPR), true negative rate (TNR), positive predictive value (PPV), and volume ratio (VR) on the testing dataset (n=14). Metrics shown = mean ± 1 standard error.

**Supplementary Table 3**

Testing Volume and MFI Accuracy and Reliability

|  | **Volume (ml)** | | | | | | | | |
| --- | --- | --- | --- | --- | --- | --- | --- | --- | --- |
| Muscle | Mean | Bias | 95% LA | MAE | RMSE | R^2^ | ICC_2,1_ | 95% CI | p |
| GMin | | | | | | | | | |
| Left | 105.7 ± 6.2 | 6.8 | -5.3 – 18.9 | 7.8 | 9.2 | 0.818 | 0.925 | 0.44 – 0.98 | p < 0.001 |
| Right | 103.0 ± 7.3 | 2.4 | -9.9 – 14.6 | 4.8 | 6.7 | 0.918 | 0.968 | 0.90 – 0.99 | p < 0.001 |
| GMed | | | | | | | | | |
| Left | 323.6 ± 20.6 | 3.0 | -26.9 – 33.0 | 10.9 | 15.6 | 0.962 | 0.982 | 0.95 – 0.99 | p < 0.001 |
| Right | 329.4 ± 23.5 | 0.0 | -50.5 – 50.6 | 18.2 | 25.8 | 0.921 | 0.962 | 0.89 – 0.99 | p < 0.001 |
| GMax | | | | | | | | | |
| Left | 890.9 ± 51.5 | 0.9 | -32.0 – 33.8 | 15.2 | 16.8 | 0.993 | 0.997 | 0.99 – 1.00 | p < 0.001 |
| Right | 886.7 ± 56.4 | -10.1 | -48.7 – 28.4 | 17.8 | 22.1 | 0.989 | 0.995 | 0.98 – 1.00 | p < 0.001 |
| TFL | | | | | | | | | |
| Left | 74.4 ± 6.1 | -3.3 | -11.7 – 5.2 | 4.2 | 5.4 | 0.953 | 0.976 | 0.89 – 0.99 | p < 0.001 |
| Right | 77.3 ± 6.0 | -2.7 | -10.7 – 5.4 | 3.1 | 4.9 | 0.964 | 0.981 | 0.92 – 0.99 | p < 0.001 |
|  | **MFI (%)** | | | | | | | | |
| Muscle | Mean | Bias | 95% LA | MAE | RMSE | R^2^ | ICC_2,1_ | 95% CI | p |
| GMin | | | | | | | | | |
| Left | 10.7 ± 1.1 | -0.6 | -2.7 – 1.5 | 0.7 | 1.2 | 0.940 | 0.967 | 0.89 – 0.99 | p < 0.001 |
| Right | 11.9 ± 1.3 | -0.7 | -1.8 – 0.4 | 0.7 | 0.9 | 0.975 | 0.987 | 0.83 – 1.00 | p < 0.001 |
| GMed | | | | | | | | | |
| Left | 11.2 ± 1.3 | -0.6 | -2.7 – 1.5 | 0.7 | 1.2 | 0.955 | 0.975 | 0.92 – 0.99 | p < 0.001 |
| Right | 10.7 ± 1.3 | -0.8 | -3.4 – 1.8 | 1.0 | 1.6 | 0.933 | 0.963 | 0.86 – 0.99 | p < 0.001 |
| GMax | | | | | | | | | |
| Left | 15.4 ± 1.7 | -0.7 | -2.1 – 0.7 | 0.7 | 1.0 | 0.979 | 0.989 | 0.90 – 1.00 | p < 0.001 |
| Right | 13.4 ± 1.7 | -0.8 | -2.1 – 0.6 | 0.8 | 1.0 | 0.976 | 0.988 | 0.87 – 1.00 | p < 0.001 |
| TFL | | | | | | | | | |
| Left | 15.3 ± 1.5 | -1.4 | -5.7 – 3.0 | 1.8 | 2.6 | 0.856 | 0.919 | 0.73 – 0.97 | p < 0.001 |
| Right | 15.7 ± 1.3 | -1.0 | -5.3 – 3.3 | 1.4 | 2.4 | 0.845 | 0.911 | 0.74 – 0.97 | p < 0.001 |

Table 3. CNN MFI and volume accuracy and reliability. Accuracy and reliability of the CNN with respect to the ground truth for muscle volume (ml) and muscle fat infiltration (MFI) measures was assessed on the testing dataset (n=14) (Fig. 3, 4). Mean=mean CNN measure±1 standard error. Bias=mean difference between CNN and ground truth. LA=limits of agreement. MAE=mean absolute error. RMSE=root mean squared error.

**Supplementary Fig 1**

**
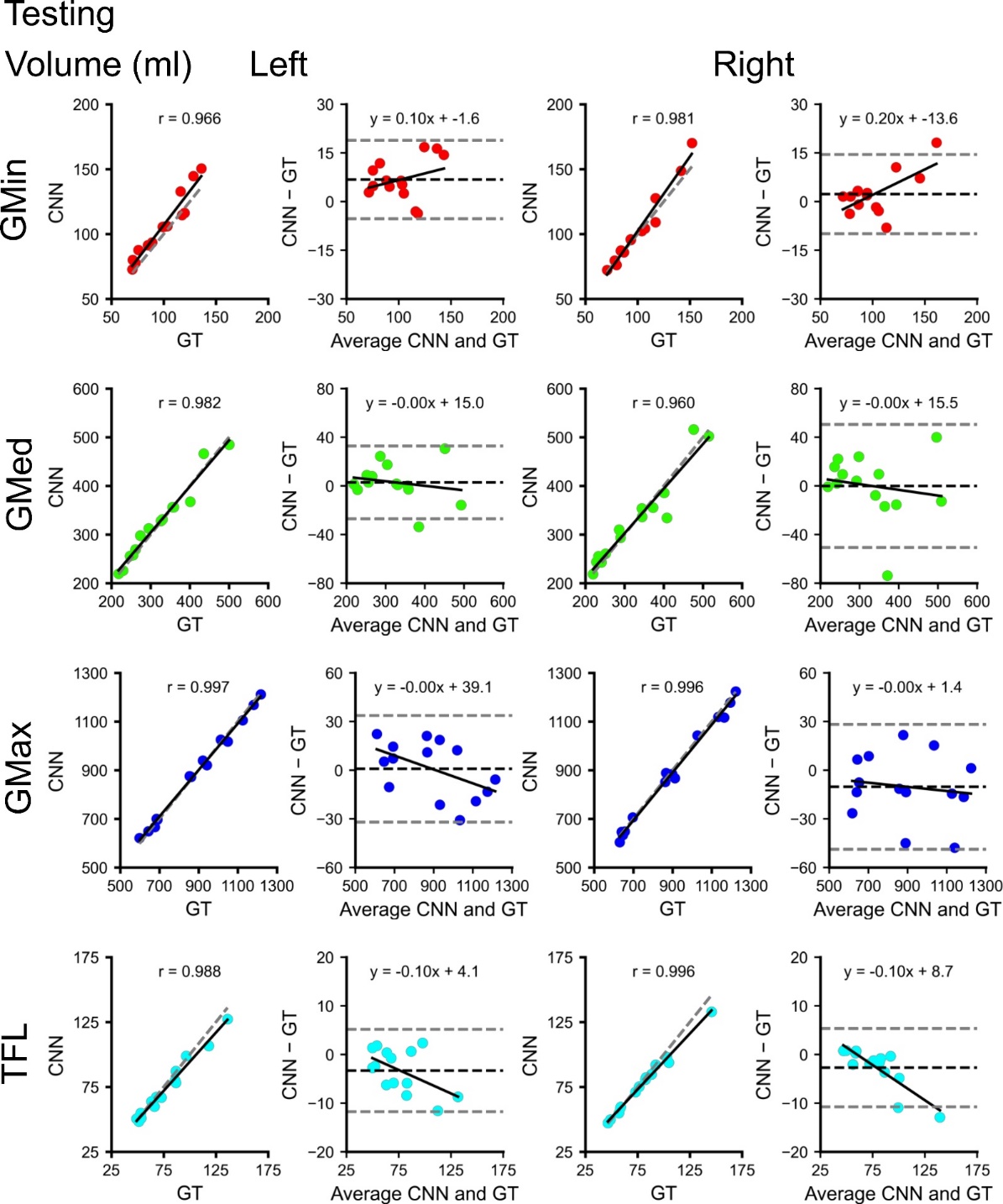
**

Supp Fig 1. Reliability and accuracy of the convolutional neural network (CNN) volume measures (ml) with respect to the ground truth (GT) on the testing dataset (n=14). Correlation and Bland-Altman plots are shown for each of the muscles. In the correlation plots, the solid black line represents the best fit line, and the dashed grey line represents perfect prediction (CNN = GT). In the Bland-Altman plots, the dashed black and grey lines indicate the bias (mean error) and the 95% limits of agreement (mean error ± 1.96 × standard deviation), respectively.

**Supplementary Fig 2**

**
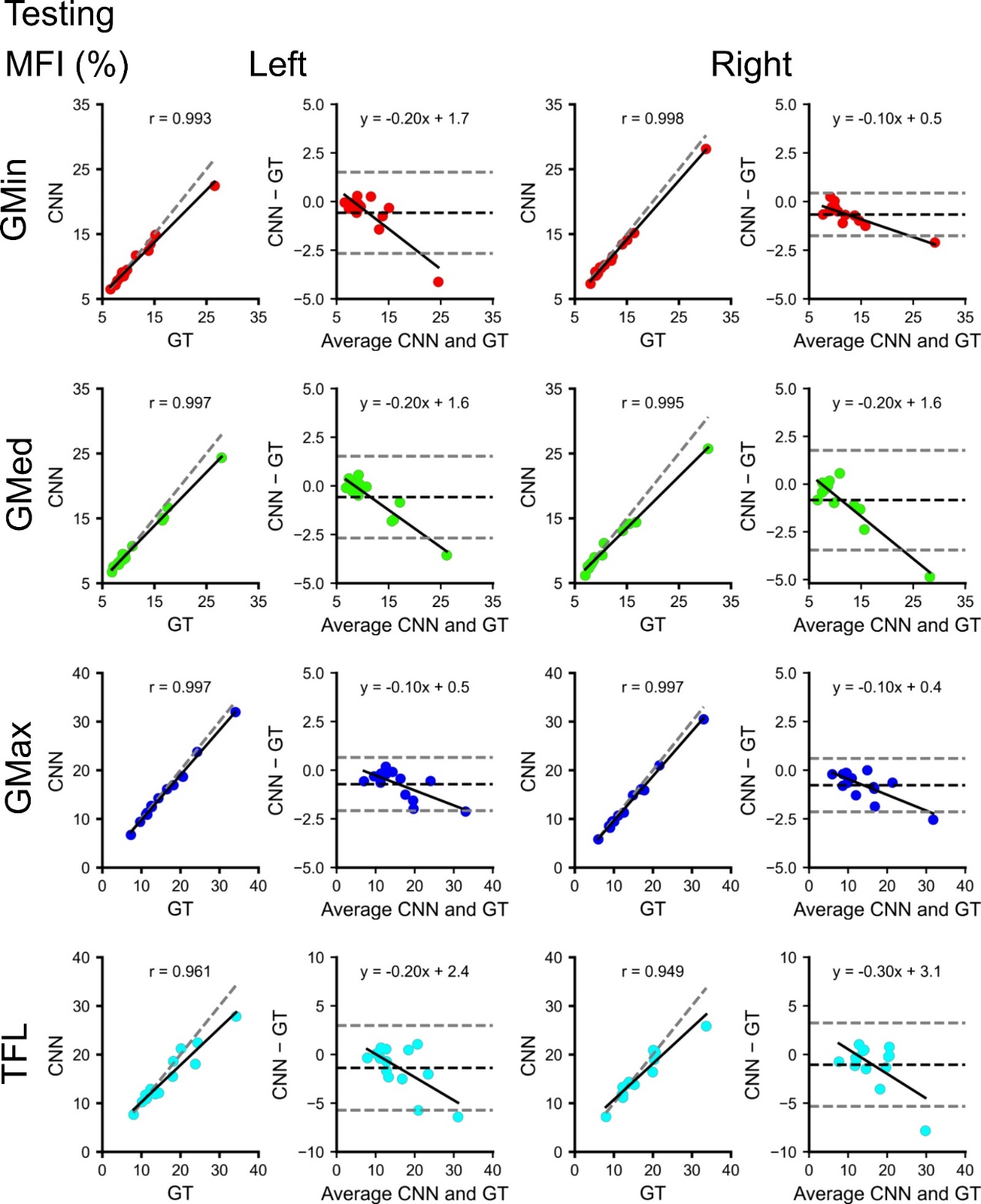
**

Supp Fig 2. Reliability and accuracy of the convolutional neural network (CNN) muscle fat infiltration (MFI) measures (%) with respect to the ground truth (GT) on the testing dataset (n=14). Correlation and Bland-Altman plots are shown for each of the muscles. In the correlation plots, the solid black line represents the best fit line, and the dashed grey line represents perfect prediction (CNN = GT). In the Bland-Altman plots, the dashed black and grey lines indicate the bias (mean error) and the 95% limits of agreement (mean error ± 1.96 × standard deviation), respectively.
